# ArtinM Mediates Murine T Cell Activation and Induces Cell Death in Jurkat Human Leukemic T Cells

**DOI:** 10.3390/ijms18071400

**Published:** 2017-06-30

**Authors:** Thiago Aparecido da Silva, Patrícia Kellen Martins Oliveira-Brito, Thiago Eleutério Gonçalves, Patrícia Edivânia Vendruscolo, Maria Cristina Roque-Barreira

**Affiliations:** Departamento de Biologia Celular e Molecular e Bioagentes Patogênicos, Faculdade de Medicina de Ribeirão Preto, Universidade de São Paulo, Ribeirão Preto, SP 14049-900, Brazil; sthiagoap@gmail.com/sthiagoap@usp.br (T.A.d.S.); patriciakellen04@hotmail.com (P.K.M.O.-B.); thiago.eg61@gmail.com (T.E.G.); pvendruscolo@usp.br (P.E.V.)

**Keywords:** CD4^+^ and CD8^+^ T cells, Jurkat T cells, lectins, ArtinM, immunomodulation

## Abstract

The recognition of cell surface glycans by lectins may be critical for the innate and adaptive immune responses. ArtinM, a d-mannose-binding lectin from *Artocarpus heterophyllus*, activates antigen-presenting cells by recognizing TLR2 *N*-glycans and induces Th1 immunity. We recently demonstrated that ArtinM stimulated CD4^+^ T cells to produce proinflammatory cytokines. Here, we further studied the effects of ArtinM on adaptive immune cells. We showed that ArtinM activates murine CD4^+^ and CD8^+^ T cells, augmenting their positivity for CD25, CD69, and CD95 and showed higher interleukin (IL)-2 and interferon (IFN)-γ production. The CD4^+^ T cells exhibited increased T-bet expression in response to ArtinM, and IL-2 production by CD4^+^ and CD8^+^ T cells depended on the recognition of CD3εγ-chain glycans by ArtinM. The ArtinM effect on aberrantly-glycosylated neoplastic lymphocytes was studied in Jurkat T cells, in which ArtinM induced IL-2, IFN-γ, and IL-1β production, but decreased cell viability and growth. A higher frequency of AnnexinV- and propidium iodide-stained cells demonstrated the induction of Jurkat T cells apoptosis by ArtinM, and this apoptotic response was reduced by caspases and protein tyrosine kinase inhibitors. The ArtinM effects on murine T cells corroborated with the immunomodulatory property of lectin, whereas the promotion of Jurkat T cells apoptosis may reflect a potential applicability of ArtinM in novel strategies for treating lymphocytic leukemia.

## 1. Introduction

Most mammalian cellular and membrane-bound proteins are glycosylated, implying that carbohydrates have an essential role in determining the functions of proteins and cells [1]. Interactions involving some cell surface glycans are especially critical for several functions of the immune system [2,3,4,5,6,7] and glycan-binding proteins are strongly associated with both innate and adaptive immunity [8]. Proteins that recognize and bind specific carbohydrate structures are classified as lectins [9], and many of them exert a lymphoproliferative activity that depends on their carbohydrate recognition domain (CRD) [10]. The CRD of lectins also participates in regulating the activation of adaptive immune cells [11,12].

T lymphocytes are fundamental protagonists of the adaptive immune system. CD4^+^ T cells play critical roles such as regulating macrophage function, helping B cells to produce antibodies, orchestrating immune responses against pathogenic microorganisms, and enhancing and maintaining CD8^+^ T cell responses [13,14]. In turn, CD8^+^ T cells promote the cytolysis of antigen-expressing target cells to protect the host against intracellular pathogens favoring the repair of injured tissue [15]. The development of effector and memory CD8^+^ T cells often depends on activated CD4^+^ T cells [16,17], which show a developmental plasticity regarding their T helper (Th) function. Th1 cells express T-bet and produce interferon (IFN)-γ as their signature cytokine, whereas Th2 cells express GATA-3 and characteristically produce interleukin (IL)-4. The differentiation of CD4^+^ T cells into either the Th1 or Th2 subset requires IL-2 production and CD25 (IL-2 receptor) expression [14,18,19,20]. Other relevant CD4^+^ T cell populations are Th17 cells, which express the transcription factor RORγt and produce IL-17A, and regulatory T cells (Tregs), which express FoxP3 and regulate the activation of T cells [20]. The differentiation of CD8^+^ T cells into cytotoxic T lymphocytes (CTLs) is associated with the up-regulation of CD69. Functional CTLs exhibit membrane-bound cytoplasmic granules that contain perforin and granzyme, whose function is to kill other cells. In addition, CTLs produce IFN-γ and tumor necrosis factor (TNF)-α, which favor the innate and adaptive immune response against intracellular pathogens [21]. Remarkably, the activation and death of T cells are tightly controlled, and CD95 (also known as Fas antigen from the tumor necrosis factor receptor superfamily) mediates signals leading to apoptotic death, maintaining both the efficacy of the immune response and the prevention of autoimmunity after T cell receptor (TCR) stimulation [22]. The expression of mature TCR is high in Jurkat T cells, which display low levels of CD4 and no CD8 [23].

Using murine spleen cells, it was previously verified [24] that the immunomodulatory lectin ArtinM exerts a lymphoproliferative activity. ArtinM, which can be obtained from the seeds of *Artocarpus heterophyllus* [25], is a homotetrameric lectin containing in each polypeptide chain a CRD with affinity to Manα1–3[Manα1–6]Man, which constitutes the core N-linked oligosaccharide structure. ArtinM induces the production of proinflammatory cytokines, which is triggered by its interactions with glycotargets on the surface of immune cells, namely neutrophils [25,26,27,28], macrophages, dendritic cells [29], and mast cells [30]. The effect of ArtinM on antigen-presenting cells (APCs), which is initiated by the recognition of TLR2 *N*-glycans by ArtinM, is responsible for promoting IL-12 production [31,32,33]. This event was considered to be central to the immunomodulatory activity exerted by ArtinM on the development of Th1 cells following the in vivo administration of lectin [31,32], resulting in an enhanced resistance to intracellular pathogens [29,31,32,34,35,36].

The notion that immunomodulation by ArtinM is exclusively due to its interaction with innate immune cells has changed after the description of its ability to activate T lymphocytes directly [24]. Besides inducing lymphoproliferation and IL-2 production [24], ArtinM promotes IL-17 release by acting directly on CD4^+^ T cells through an interaction with CD3 [37]. We designed the present study to further investigate the effects of ArtinM on murine T cells and search for new mechanisms accounting for the Th1-inducing property of lectins. Additionally, we examined the effect of ArtinM on aberrantly-glycosylated lymphocytes using the Jurkat human T leukemia cell line. We showed that ArtinM exerts direct effects on CD4^+^ T cells and CD8^+^ T cells that conceptually contribute to its immunomodulatory activity towards a Th1 response. In contrast, ArtinM promoted the cell death of Jurkat T cells, which are neoplastic lymphocytes.

## 2. Results

### 2.1. ArtinM Induces the Activation of Murine CD4^+^ and CD8^+^ T Cells

**Figure 1:**
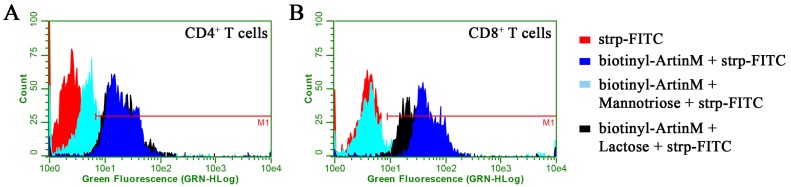
ArtinM binding to the surface of CD4^+^ and CD8^+^ T cells is specifically sugar-inhibited. Cells were isolated from a suspension of C57BL/6 mice spleen cells. After fixation, they were incubated with biotinylated ArtinM (25 µg/mL) for 40 min (**blue**). For some indicated experiments, ArtinM was pre-incubated with mannotriose (2 mM; **cyan**) or lactose (20 mM; **black**) for 40 min, and then added to CD4^+^ (**A**) or CD8^+^ (**B**) T cells suspensions for an additional 40 min incubation. After washing the cells, they were analyzed to determine the presence of bound ArtinM through reaction with streptavidin-fluorescein isothiocyanate (FITC) (5 µg/mL). Streptavidin-FITC alone (**red**) was used as the negative control. The cells were analyzed by flow cytometry for cell counting and green fluorescence intensity measurement. The red line (M1) indicates the cells that were positive for ArtinM binding.

We reported previously that ArtinM binds to the surface of murine spleen cells through carbohydrate recognition [24]. Herein, we studied the interaction of ArtinM with particular T cell populations. We verified that ArtinM binds equivalently to the surfaces of purified CD4^+^ and CD8^+^ T cells in a manner that is dependent on its carbohydrate recognition property, since the pre-incubation of ArtinM with its specific ligand Manα1–3[Manα1–6]Man inhibited its interactions with both CD4^+^ and CD8^+^ T cells. In contrast, lactose, a disaccharide that is not specific for ArtinM, exerted no significant effect on the lectin binding to the assayed cells, although the mean fluorescence intensity (MFI) provided by CD8^+^ T cells has slightly shifted (Figure 1), a fact that deserves further studies.

**Figure 2:**
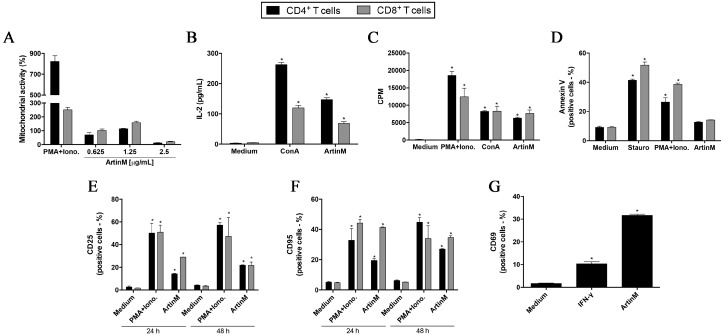
ArtinM stimulates CD4^+^ and CD8^+^ T cells activation. CD4^+^ and CD8^+^ T cells (1.5 × 10^6^/mL) were purified from a suspension of C57BL/6 mice spleen cells distributed in 96-well microplates and incubated at 37 °C, for the indicated periods, under stimulation with ArtinM at different concentrations (**A**) or with 1.25 μg/mL ArtinM (**B**–**G**). Concanavalin A (ConA; 5 μg/mL), phorbol 12-myristoyl 13-acetate (PMA; 50 ng/mL) plus ionomycin (1 μM), and staurosporine (Stauro; 1 μM), or interferon-γ (IFN-γ) (50 ng/mL) were used as positive controls, as indicated in each respective panel. Culture medium alone was used as the negative control. (**A**) After 24 h of incubation, 3-(4,5-dimethyl-thiazol-2-yl)-2,5-diphenyl-tetrazolium bromide (MTT) was added to the culture medium. The mitochondrial activity was measured through MTT reduction and expressed as the absorbance relative to that of the negative control as a percentage value; (**B**) the cell culture supernatants were analyzed for their IL-2 levels through an enzyme-linked immunosorbent assay (ELISA); (**C**) after 48 h of stimulation, [^3^H]-thymidine (0.5 μCi/well) was added. Following a further 12 h of incubation, cell proliferation was measured by detecting [^3^H]-thymidine incorporation (expressed as counts per minute); (**D**–**G**) after 24 or 48 h of stimulation, CD4^+^ and CD8^+^ T cells were stained with Annexin V-FITC, anti-CD25 phycoerythrin (PE), anti-CD95 FITC, or anti-CD69 FITC. The frequency of stained cells was analyzed by flow cytometry. The results are expressed as means ± standard error of the mean (SEM); * *p* < 0.05 compared to the negative control (medium alone).

To investigate whether ArtinM binding triggers the activation of either CD4^+^ or CD8^+^ T cells, we first assessed their mitochondrial activity following 24 h stimulation with ArtinM. The MTT assay results showed that ArtinM treatment at concentrations of 0.625 and 1.25 μg/mL increased the mitochondrial activities of CD4^+^ and CD8^+^ T cells. The maximum activity was observed after treatment with 1.25 μg/mL ArtinM, while a higher ArtinM concentration (2.5 μg/mL) substantially reduced the mitochondrial activity, suggesting that it was toxic for both cell populations (Figure 2A). The capacity of ArtinM to induce the activation of CD4^+^ and CD8^+^ T cells was additionally assessed by measuring IL-2 production and cell proliferation. We found high IL-2 levels in the supernatants of CD4^+^ and CD8^+^ T cells that were stimulated with 1.25 μg/mL ArtinM for 24 h (Figure 2B). This stimulus also induced the proliferation of both cell populations (Figure 2C), as estimated through the ^3^H-deoxythymidine ([_3_H]-TdR) incorporation assay. Then, the stimulated cells were Annexin V-stained and analyzed by flow cytometry. The percentage of cells that were positive for Annexin V staining was similar among stimulated and non-stimulated CD4^+^ and CD8^+^ T cells (Figure 2D), whereas stimulation with staurosporine or phorbol 12-myristate 13-acetate (PMA) plus ionomycin promoted a high incidence of apoptotic CD4^+^ and CD8^+^ T cells. We concluded that the stimulation of T cells with ArtinM did not induce cell death at low concentrations.

### 2.2. CD4^+^ and CD8^+^ T Cells Activation Marker Expression Is Induced by ArtinM

To better characterize the induction of T cell activation by ArtinM, we studied the expression of CD25 (α chain of the IL-2 receptor) and CD95 (a member of the tumor necrosis factor receptor superfamily) on CD4^+^ and CD8^+^ T cells following 24 and 48 h stimulation with ArtinM. The cells were then analyzed by flow cytometry, which showed that ArtinM induced a significant increase in the frequency of CD25- and CD95-positive CD4^+^ and CD8^+^ T cells, in comparison to that in unstimulated cells (Figure 2E,F). Additionally, we examined the ArtinM-stimulated CD8^+^ T cells for the expression of CD69 (also known as very early activation antigen). ArtinM stimulation augmented the frequency of CD69-positive CD8^+^ T cells, in comparison to that in unstimulated cells (Figure 2G). These observations reinforce the idea that ArtinM promotes the activation of both CD4^+^ and CD8^+^ T cells.

### 2.3. CD4^+^ and CD8^+^ T Cells Show a Marked Proinflammatory Profile after ArtinM Stimulation

**Figure 3:**
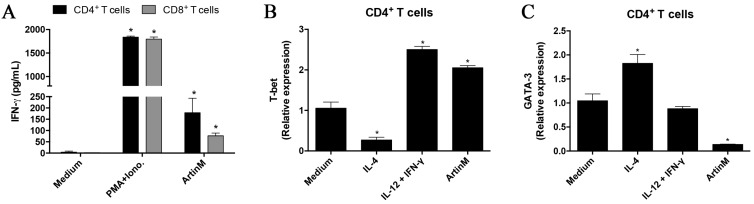
Detection of activation markers in ArtinM-stimulated CD4^+^ and CD8^+^ T cells. CD4^+^ and CD8^+^ T cells (1.5 × 10^6^/mL) were distributed in 96-well microplates and incubated under stimulation with ArtinM (1.25 μg/mL), PMA (50 ng/mL) plus ionomycin (1 μM), IL-4 (50 ng/mL), or IL-12 (50 ng/mL) plus IFN-γ (30 ng/mL) at 37 °C for different periods of time. Medium alone was used as the negative control. (**A**) Culture supernatants of CD4^+^ and CD8^+^ T cells were used to measure IFN-γ production by ELISA after 48 h of incubation; (**B**,**C**) CD4^+^ T cells were stimulated for 8 h and the extracted RNA was used for real-time quantitative polymerase chain reaction analysis of T-bet and GATA-3 mRNA. The results are expressed as means ± SEM; * *p* < 0.05 compared to the negative control (medium alone).

Because ArtinM promotes the activation of CD4^+^ and CD8^+^ T cells, we studied IFN-γ production by these cells after 48 h stimulation with ArtinM. We verified that the supernatant of the stimulated CD4^+^ and CD8^+^ T cells contained significantly higher IFN-γ levels in comparison to that of unstimulated cells (Figure 3A). To characterize the pattern of CD4^+^ T cell activation induced by ArtinM, we examined the relative expression of transcription factors related to Th1- and Th2-differentiation following 8 h stimulation with ArtinM. We verified that ArtinM stimulation was associated with significantly higher T-bet expression (Figure 3B) and lower GATA-3 expression (Figure 3C) when compared to unstimulated cells. The pro-inflammatory pattern of the response induced by ArtinM is compatible with the protective effect against intracellular pathogens exerted in vivo by lectin administration.

### 2.4. Functional Relevance of CD3 as a Glycotarget of ArtinM on CD4^+^ and CD8^+^ T Cells

**Figure 4:**
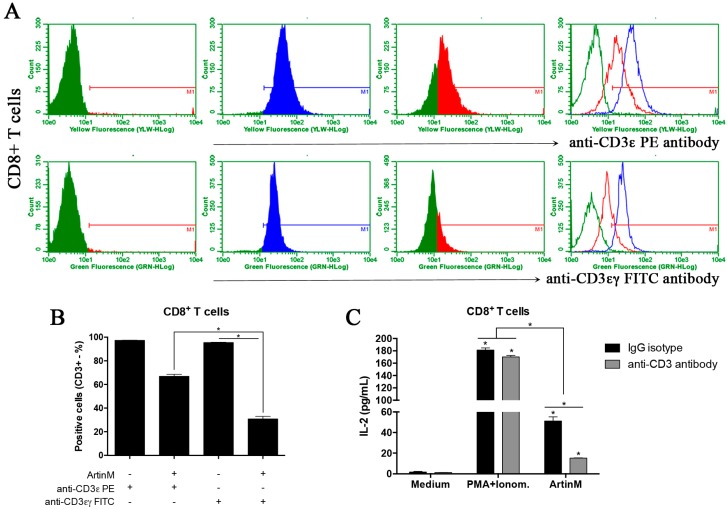
The functional effect of competition between ArtinM and anti-CD3 antibody. (**A**) Purified CD8^+^ T cells (1 × 10^6^/mL) were fixed and incubated with or without ArtinM (25 µg/mL) for 40 min. After washing, the cells were incubated for 40 min with 145-2C11 monoclonal antibody (conjugated to PE; upper panel) or 17A2 monoclonal antibody (conjugated to FITC; lower panel), which are specific to the CD3ε chain and CD3εγ chains, respectively. The labeled cells were analyzed by flow cytometry, and the histograms represent the percentage of positive cells for anti-CD3 antibody after preincubation with (**red**) or without (**blue**) ArtinM. As shown in panel (**B**), the replicates and the overlay represent all these conditions; the statistical analysis was performed using Dunn’s multiple comparison test. (**C**) Purified CD8^+^ T cells (1.5 × 10^6^/mL) were incubated with 17A2 monoclonal antibody against CD3 receptor (8 µg/mL; gray bar) or IgG isotype control (8 μg/mL, black bar), for 40 min at 4 °C. The cells were incubated for 48 h at 37 °C under stimulation with ArtinM (1.25 µg/mL), PMA (50 ng/mL) plus ionomycin (1 µM), or medium alone, and the culture supernatants were used to determine the IL-2 levels by ELISA. The results are expressed as means ± SEM; * *p* < 0.05 indicates a significant difference.

We previously reported that the ArtinM-induced activation of CD4^+^ T cells depends on its interaction with CD3. We based this statement on our findings that ArtinM significantly reduced the labeling of CD4^+^ T cells with the anti-CD3 antibody, and the anti-CD3 antibody blocked the effects of ArtinM on IL-2- and IL-17-production by CD4^+^ T cells [24,37]. Similar procedures were adopted in the current work to study CD8^+^ T cells. First, the isolated cells, preincubated with or without ArtinM, were analyzed by flow cytometry to determine the frequency of CD3-stained cells. The CD3 staining was performed by using two different monoclonal antibodies, one derived from the clone 145-2C11, which recognizes the mouse CD3ε-chain, and the other from the clone 17A2, recognizing mouse CD3εγ-chains. The pre-incubation of CD8^+^ T cells with ArtinM caused a 3.5-fold decrease in the frequency of CD3εγ-chains-labeled cells and a 1.5-fold reduction in the frequency of CD3ε-chain-labeled cells (Figure 4A,B). Concerning the frequency of CD4^+^ T cells stained with the same antibodies, da Silva et al. [24] reported that pre-incubation with ArtinM drastically reduced the frequency of cells labeled with the anti-CD3εγ-chains antibody. In a similar context, we verified herein that after preincubation with ArtinM, only a slight reduction in the frequency of CD4^+^ T cells stained for CD3ε-chain occurred (Figure S1). To determine whether the CD3εγ-chains contain the ArtinM glycotarget on CD4^+^ and CD8^+^ T cells, we investigated whether the anti-CD3εγ-chains antibody (17A2 monoclonal) could affect the IL-2 production by CD8^+^ T cells in response to ArtinM. Interestingly, we verified that treatment with the anti-CD3εγ-chains antibody resulted in a 70% inhibition of the ArtinM-induced IL-2 release by CD8^+^ T cells (Figure 4C). These findings suggest that the CD3εγ chains contain a functionally relevant glycotarget for ArtinM, whose recognition triggers the activation of both CD4^+^ and CD8^+^ T cells.

### 2.5. The Interaction of ArtinM with Jurkat T Cells Results in IL-2 Production

**Figure 5:**
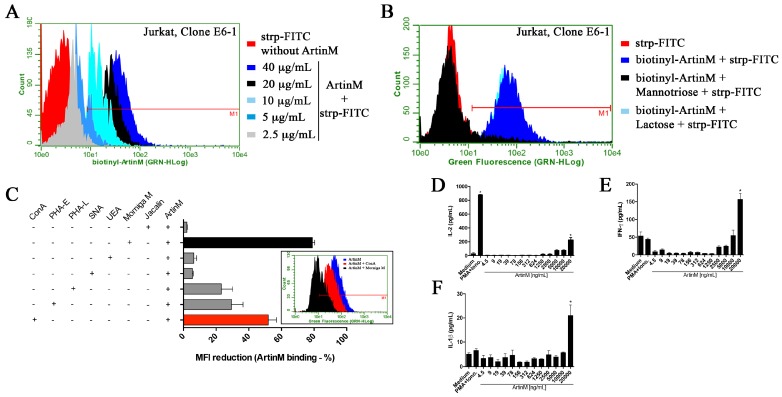
ArtinM-binding on Jurkat T cells and cytokine production by ArtinM-stimulated Jurkat T cells. Jurkat T cells (2 × 10^5^/mL) were fixed and incubated for 40 min with different treatments, as follows: (**A**) non-pre-treated biotinylated ArtinM (2.5–40 μg/mL); (**B**) biotinylated ArtinM (40 µg/mL) that was pre-incubated with mannotriose (2 mM) or lactose (20 mM) for 40 min; or (**C**) biotinylated ArtinM (40 µg/mL) concomitantly with another lectin (ConA, phytohemagglutinin [PHA]-E, PHA-L, *Sambucus nigra* lectin [SNA], *Ulex europaeus* agglutinin [UEA], Morniga M, or Jacalin), also at 40 µg/mL. Then, the cells were washed and the ArtinM binding was revealed by reaction with streptavidin-FITC (5 µg/mL) followed by analysis by flow cytometry. Streptavidin-FITC alone was used as the negative control. (**A**–**C**) The histograms represent the fluorescence intensity of the cells that were positive for ArtinM binding, as indicated by the red line (M1), and the graph in **C** shows the mean fluorescence intensity (MFI) reduction (%) of ArtinM binding to Jurkat T cells in the presence of each lectin; (**D**–**F**) Jurkat T cells (2 × 10^5^/mL) were stimulated with ArtinM (4.5–20,000 ng/mL) or PMA (50 ng/mL) plus ionomycin (1 μM) for 48 h, and the culture supernatants were used to quantify the levels of IL-2, IFN-γ, and IL-1β by ELISA. The results are expressed as means ± SEM; * *p* < 0.05 compared to the negative control (medium alone).

The effects of ArtinM on murine T cells prompted us to extend the investigation to human T cells. We chose to work with the Jurkat cell line, realizing that these cells are derived from leukemic cells and could have altered glycosylation because of the neoplastic process. First, we assayed the binding of biotinylated ArtinM to Jurkat T cells in the presence or absence of mannotriose. A flow cytometric analysis showed that ArtinM bound to Jurkat T cells in a dose-dependent manner, establishing an interaction that was inhibited by pre-incubation of ArtinM with mannotriose (Figure 5A,B). ArtinM binding was also examined after the Jurkat T cells were incubated with several lectins having diverse specificities for carbohydrate recognition, as follows: ConA (α-Manα1–2Man); PHA-E (Galβ1–4GlcNAcβ1–2Manα1–6[GlcNAcβ1–4]Man); PHA-L (Galβ1–4GlcNAcβ1–6[Galβ1–4GlcNAcβ1–2]Man); SNA (Neu5Acα1–6Gal); Jacalin (Galβ1–3GalNAc); UEA (Fucα1–3); and Morniga M (Manα1–6[Manα1–3]Man). We verified that ConA and Morniga M inhibited the interaction of ArtinM with Jurkat T cells by 50 and 80%, respectively (Figure 5C). Intermediate inhibition was shown by PHA-E (29.3%) and PHA-L (23.2%), whereas Jacalin, UEA, and SNA had no significant effect on the binding of ArtinM to Jurkat cells. These results suggest that ConA and Morniga M bind to *N*-glycans that are at least partially coincident with those targeted by ArtinM on Jurkat T cells.

To investigate whether ArtinM activates Jurkat T cells, we measured the IL-2 levels in the culture supernatant after 48 h stimulation with ArtinM. Treatment with ArtinM induced IL-2 production, but only at 20 µg/mL (Figure 5D), and ArtinM also induced the production of IFN-γ and IL-1β by Jurkat T cells, but again high lectin concentrations were required (Figure 5E,F).

### 2.6. ArtinM Induces the Apoptosis of Jurkat T Cells

**Figure 6:**
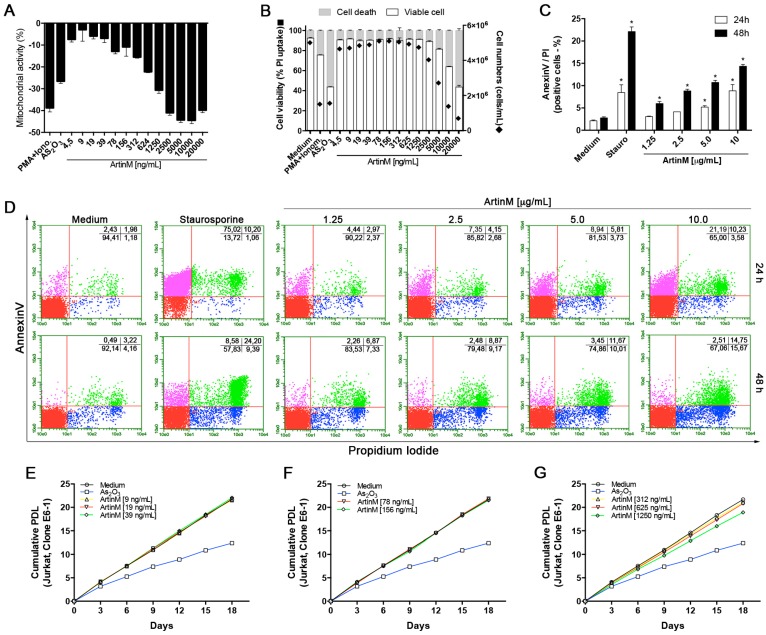
The effect of ArtinM on the viability of Jurkat T cells. (**A**,**B**) Jurkat T cells (1 × 10^5^/mL) were distributed in a 96-well microplate and cultured for 48 h under ArtinM stimulation (4.5–20,000 ng/mL). PMA (50 ng/mL) plus ionomycin (1 µM), and As_2_O_3_ (3 μM), were used as positive controls. Medium alone was used as the negative control. (**A**) MTT was added to the culture medium to measure mitochondrial activity, which was expressed as the absorbance relative to that of the negative control as a percentage value; (**B**) Determination of the percentages of viable and dead cells through measuring propidium iodide (PI) uptake by flow cytometry; cell concentrations (cells/mL) were counted using a Neubauer chamber; (**C**,**D**) Jurkat T cells (1 × 10^5^/mL) were stained with Annexin V-FITC and PI at 24 and 48 h after stimulation with ArtinM (1.25–10 μg/mL) or the positive control, staurosporine (1 µM). Culture medium alone was used as the negative control. The frequency of double-stained cells (Annexin V-FITC and PI) was determined by flow cytometry (dot plot; **D**) and expressed as the percentage of positive cells (**C**); (**E**–**G**) The population doubling level assay was used to determine the cumulative cell growth of Jurkat T cells for 18 days in the presence of ArtinM (9–1250 ng/mL), As_2_O_3_ (1.5 μM), or medium alone. The cumulative population doubling level was determined every 72 h as described in the Materials and Methods. Results are expressed as means ± SEM; * *p* < 0.05 compared to the negative control (medium alone).

We reported previously that ArtinM exerted a toxic effect on murine spleen cells in a concentration-dependent manner [24]. Herein, we observed that CD4^+^ and CD8^+^ T cells showed a reduced mitochondrial activity when stimulated with 2.5 µg/mL ArtinM (Figure 2A). Then, we investigated the mitochondrial activity of Jurkat T cells under stimulation with ArtinM at different concentrations (0.0045–20 µg/mL). ArtinM treatment resulted in decreased mitochondrial activity. Reductions in mitochondrial activity of at least 10% were caused by ArtinM concentrations ≥0.078 µg/mL. Decreases of more than 40% were observed at ArtinM concentrations ≥2.5 µg/mL. The positive controls of PMA plus ionomycin and arsenic trioxide (As_2_O_3_) for cell activation and cell death, respectively, were associated with 39% and 25% reductions of mitochondrial activity (Figure 6A). To explore this effect of ArtinM on Jurkat T cells, we determined the frequency of cells that incorporated PI or were Annexin V-stained after 24 and 48 h stimulation with ArtinM. ArtinM concentrations ≥2.5 µg/mL promoted reductions in the frequency of viable Jurkat T cells (Figure 6B). In this context, Jurkat T cells that were stimulated for 48 h with ArtinM (1.25–10 µg/mL) showed a high frequency of staining with Annexin V and PI. The percentage of positive cells for both Annexin V and PI was identified by flow cytometry, which verified that all concentrations of ArtinM induced the apoptosis of Jurkat T cells (Figure 6C,D). To evaluate whether ArtinM at concentrations lower than 1.25 µg/mL could affect the growth of Jurkat T cells, we performed a cumulative PDL assay for 18 days, as described in the Materials and Methods. The results indicated that ArtinM did not promote the growth of Jurkat T cells compared with culture medium alone (Figure 6E–G), and As_2_O_3_ was used as a positive control for growth inhibition. These results demonstrate that ArtinM promotes the apoptosis of Jurkat T cells.

### 2.7. ArtinM-Induced Apoptosis of Jurkat T Cells Is Mediated by Caspases

**Figure 7:**
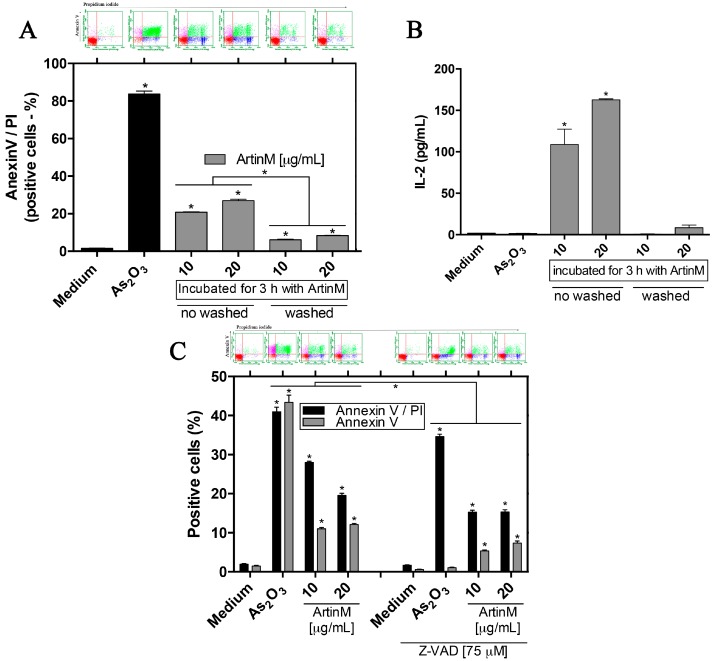
A mechanism involved in the apoptosis of ArtinM-stimulated Jurkat T cells. Cells (2 × 10^5^/mL) were distributed in a 96-well microplate and cultured for 24 or 48 h in the presence of ArtinM (10 or 20 μg/mL) or As_2_O_3_ (3 μM). Medium alone was used as negative control. Jurkat T cells were stained with Annexin V-FITC and PI. The frequency of double-stained cells (Annexin V/PI) or single-stained cells (Annexin V) was determined by flow cytometry (dot plot; above) and expressed as the percentage of positive cells. (**A**) Jurkat T cells were incubated with ArtinM for 3 h. Then, the cells were washed, or not, to remove the lectin contained in the culture supernatant. Afterwards, the cells were cultured for additional 21 h and stained with Annexin V-FITC and PI. IL-2 production was quantified in the cell supernatant by ELISA (**B**); (**C**) A broad-spectrum caspase inhibitor (Z-VAD; 75 μM) was added or not to a Jurkat T cells suspension for 3 h, then the cells were stimulated with ArtinM, As_2_O_3_, or medium alone. The results are expressed as means ± SEM; * *p* < 0.05 compared to the negative control (medium alone) or between treatment groups.

We hypothesized that the continued presence of ArtinM for 24 or 48 h in the Jurkat T cell cultures could create a bias due to its possible interaction with glycoproteins that are responsible for triggering apoptosis, such as death receptors. Therefore, following incubation with ArtinM for 3 h, Jurkat T cells were washed to remove the lectin, cultured for an additional 21 h, and analyzed for the frequency of Annexin V/PI-stained cells and IL-2 production. We verified that the frequency of Annexin V/PI-stained cells following ArtinM treatment for 3 h was higher than that in unstimulated cells, but significantly lower than that in the cells treated with ArtinM for 21 h (Figure 7A). Furthermore, the IL-2 production by Jurkat T cells that were stimulated with ArtinM for 3 h was similar to that by unstimulated cells (Figure 7A, inset). These observations indicate that prolonged ArtinM stimulation in Jurkat T cell cultures promotes cell apoptosis more than a shorter duration of ArtinM stimulation does.

To investigate the mechanism of ArtinM-induced death in Jurkat T cells, we studied the role of caspases using a broad-spectrum caspase inhibitor (Z-VAD). The Jurkat T cells were incubated for 3 h with or without 75 μM Z-VAD, and cultured for an additional 45 h under ArtinM stimulation. The ArtinM-induced apoptosis decreased significantly after the inhibition of caspases (Figure 7C). The effect of As_2_O_3_ (positive control) on cell death also decreased when caspases were inhibited (Figure 7C). Therefore, caspases participate in the Jurkat T cells apoptosis induced by ArtinM.

### 2.8. Inhibitors of PKC, JNK, p38 MAPK, and ERK Do Not Block the ArtinM-Induced Apoptosis of Jurkat T Cells

**Figure 8:**
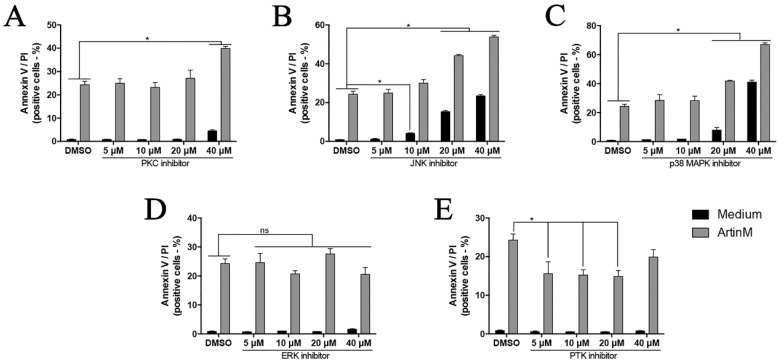
Effect of inhibitors against PKC, JNK, p38 MAPK, ERK, and PTKs on the apoptosis of Jurkat T cells induced by ArtinM. Cells (2 × 10^5^/mL) were distributed in a 96-well microplate and cultured for 210 min in the presence or absence of the following pharmacological inhibitors at the indicated concentrations: (**A**) H-7 (PKC inhibitor); (**B**) SP600125 (JNK inhibitor); (**C**) SB202190 (p38 MAPK inhibitor); (**D**) PD98059 (ERK inhibitor); and (**E**) genistein (PTKs inhibitor). Then, the cells were stimulated for 48 h with ArtinM (20 μg/mL), As_2_O_3_ (3 μM), or medium alone. Finally, the cells were analyzed by flow cytometry for Annexin V-FITC binding and PI incorporation. Double-positive cells for Annexin V/PI were determined following stimulation with ArtinM (gray bar) or medium alone (black bar) after the treatments with each inhibitor. Dot plots showing the percentage of positive cells for Annexin V/PI or Annexin V under different experimental conditions are presented in Figure S1. The results are expressed as means ± SEM; * *p* < 0.05 compared to the negative control group (dimethyl sulfoxide vehicle alone) under the same conditions.

The pharmacological inhibitors of the cell cycle regulation system have been extensively studied because they appear to be promising antitumor agents [38,39,40]. The inhibition of mitogen-activated protein kinases (MAPKs), protein kinase C (PKC), and protein tyrosine kinases (PTKs) has been evaluated regarding its effect on the cell cycle [40,41,42]. In this context, we investigated the effect of ArtinM on the apoptosis of Jurkat T cells in the presence of the following pharmacological inhibitors: genistein (PTKs inhibitor), PD98059 (ERK inhibitor), SB202190 (p38 MAPK inhibitor), H-7 (PKC inhibitor), and SP600125 (JNK inhibitor). The Jurkat T cells were incubated with different concentrations of either inhibitor for 210 min and subsequently incubated with or without ArtinM (20 μg/mL). After 48 h incubation, the Jurkat T cells were stained with Annexin V/PI and analyzed by flow cytometry. Among the pharmacological agents, those inhibiting PKC, JNK, p38, and ERK did not inhibit the apoptosis of Jurkat T cells induced by ArtinM (Figure 8 and Figure S2). Otherwise, under the effect of the PTKs inhibitor, the ArtinM-induced apoptosis of Jurkat T cells significantly decreased (Figure 8 and Figure S2).

## 3. Discussion

We demonstrated previously that ArtinM has a lymphoproliferative property toward murine spleen cells [24] and induces IL-17 production by CD4^+^ T cells [37]. These findings supported the present investigation on the effects of ArtinM on different T cell populations. We verified that ArtinM induces the activation of murine CD4^+^ and CD8^+^ T cells in a proinflammatory pattern. Otherwise, the ArtinM stimulation of Jurkat human leukemic T cells resulted in cell apoptosis. Therefore, our studies on the effects of ArtinM on adaptive immune cells isolated from different sources demonstrated that carbohydrate recognition by ArtinM directly influences their behavior.

The glycan-binding property of ArtinM is responsible for its interaction with spleen cells from C57BL/6 and BALB/c mice [24,37]. Herein, we demonstrated that ArtinM targeted both purified CD4^+^ and CD8^+^ T cells through its CRD. The glycan fine-specificity of ArtinM, which is a mannose-binding protein from the family of Jacalin-related lectins (JRL), was evaluated previously by different methods. Initial biochemical studies identified its ability to recognize mannopentose, mannotriose, and α-methyl-mannosides [43,44]. Then, using surface plasmon resonance analysis, Barre et al. [45] reported that the ArtinM CRD can accommodate other oligosaccharide. The crystal structures of ArtinM complexed with Manα1–3[Manα1–6Manα1–3(Manα1–6)]Manβ1–4 and Manα1–3[Manα1–6]Manβ1–4 verified that the ArtinM CRD contains a primary site and a secondary site for interaction with oligosaccharide [43]. The study on the ArtinM fine-specificity of glycan recognition was expanded by Nakamura-Tsuruta et al. [46] using frontal affinity chromatography, which showed that ArtinM binds with good affinity to the *N*-glycan core structures Manα1–3[Manα1–6]Manβ1–4 and GlcNAcβ1–2, which are added to the Manα1–6Manβ1–4 branched *N*-glycans. In the same study, a similar specificity was verified for a second Man-binding JRL, which is Morniga M. More recently, in collaboration with the group headed by Dr. Ten Feizi, we analyzed the glycan fine-specificity of ArtinM using a glycan microarray. This research confirmed the preference of the ArtinM primary site for probes having the core structure Manα1–3[Manα1–6]Manβ1–4. It also showed the contribution of the secondary site of ArtinM to enhancing the affinity of the CRD for probes containing Fucα1–6 or GlcNAcα1–2 associated with Manα1–3[Manα1–6]Manβ1–4 [47,48,49]. The core structure Manα1–3[Manα1–6]Manβ1–4 recognized by ArtinM is also targeted by Morniga M [50,51], which is consistent with the marked correlation between the glycan-binding specificities and phylogenies of ArtinM and Morniga M [46]. Interestingly, ConA, a legume lectin that is not phylogenetically close to jacalina-related lectins (JRLs), also recognizes the same trimannoside. Nevertheless, the glycan fine-specificity of ConA is directed towards high-mannose and bisected-hybrid-type *N*-glycans [52], which are not recognized by ArtinM according to the analysis performed using the glycan microarray (Figure S3). Previous studies on the effects of ConA and Morniga M on lymphocytes [50,53] showed that the recognition of *N*-glycans by these lectins accounts for the T cell responses. Interestingly, *O*-glycan-binding lectins may also induce responses in lymphocytes [54,55]. The effect of lectins on adaptive immune cells is usually approached by analyzing the occurrence of lymphoproliferative responses [56,57,58,59,60,61], which we previously verified in ArtinM-stimulated murine spleen cells [24]. Isolated CD4^+^ and CD8^+^ T cells also proliferated under ArtinM stimulation in a concentration-dependent manner. However, at high concentrations of ArtinM, we observed a suppressive effect, which was also reported to be exerted by high concentrations of ConA [53]. This toxic effect of ArtinM on murine CD4^+^ and CD8^+^ T cells occurred at concentrations higher than 1.25 μg/mL. These findings suggest that high concentrations of ArtinM might trigger activation-induced cell death, which was also verified in human T lymphocytes incubated with Morniga M or ConA [50].

We found that the optimal concentration of ArtinM for inducing cell activation did not promote the apoptosis of murine CD4^+^ and CD8^+^ T cells, but stimulated IL-2 production and high proliferation rates. These results show that ArtinM acts as an antigen-independent mitogen, a property already attributed to ConA vis-a-vis its ability to induce the proliferation of CD4^+^ and CD8^+^ T cells [62]. Interestingly, ArtinM alone induced the activation of CD4^+^ and CD8^+^ T cells, whereas *Amaranthus leucocarpus* lectin was reported to enhance the anti-CD3 antibody-mediated activation of human peripheral blood CD4^+^ and CD8^+^ T cells [55,63]. Beyond cell proliferation, ArtinM also promoted the expression of CD25, which is an activation-associated molecule, on CD4^+^ and CD8^+^ T cells. Considering that TCR stimulation alone did not induce CD25 expression in naïve CD4^+^ T cells [64], we postulate that ArtinM promotes both TCR stimulation and co-stimulation. We previously reported that the interaction of ArtinM with CD3 accounts for the induction of IL-2 and IL-17 production by CD4^+^ T cells [24,37]. In the current work, we further demonstrated that CD3 participates in the ArtinM-induced activation of CD8^+^ T cells. In addition to CD25, TCR stimulation upregulates the expression of CD69 and CD95 [22,64,65], which also occurred in ArtinM-activated CD4^+^ and CD8^+^ T cells. The Fas-associated death domain associated with CD95 allows the elimination of activated T cells through the CD95/CD95L system [66,67]. However, the ArtinM-stimulated CD4^+^ and CD8^+^ T cells were not stained by Annexin V. Novel roles have recently been attributed to CD95 that involve functions apart from cell death induction, such as acting as a silencer of the immune response [22]. Thus, CD95 may control the proliferation of CD4^+^ and CD8^+^ T cells that is stimulated by ArtinM.

It is well established that, among activated CD4^+^ and CD8^+^ T cells, those producing IFN-γ and IL-2 preferentially survive [68,69,70]. Interestingly, ArtinM induced CD4^+^ and CD8^+^ T cells to produce IFN-γ and IL-2, which are inflammatory cytokines that are known to contribute to the expansion of effector CD4^+^ and CD8^+^ T cell populations [68,71,72]. Some cytokines play a critical role in inducing the transcription factors that determine the differentiation of CD4^+^ T cells [73,74]. The effect of ArtinM on CD4^+^ T cells was associated with the overexpression of T-bet, which is a major factor for driving cell differentiation toward the Th1 axis, whose role in favoring the elimination of intracellular pathogens is well established [75]. Therefore, the effect of ArtinM on CD4^+^ and CD8^+^ T cells may contribute to modulating immunity and conferring protection against infections by intracellular pathogens, as previously reported [29,31,32,34,35,36]. The property of ArtinM described herein is novel, since it improves our understanding of the interaction between ArtinM and adaptive immune cells, and provides new mechanisms for the well-established immunomodulatory activity of ArtinM.

After elucidating the effects of ArtinM on murine CD4^+^ and CD8^+^ T cells, we became interested in evaluating its activities towards a T leukemia cell line. For that purpose, we used the Jurkat T leukemia cell line, which was cultured according to the protocol recommended by the ATCC cell biology collection. In cancer cells, changes in the glycocalyx include branching deviations of *N*-glycans, constituting alterations that impair the interaction of the cells with several lectins [76]. Consequently, we were aware that the effects of ArtinM on Jurkat T cells could differ from those exerted on murine CD4^+^ and CD8^+^ T cells. In this context, Morniga M, a mannose-specific lectin, had been described as activating T cells, but exerting toxic effects on Jurkat T cells [50]. Herein, we verified that ArtinM binds on the surface of Jurkat T cells through carbohydrate recognition and competes with ConA and Morniga M for the same *N*-glycans. Jurkat T cells were also stimulated to produce cytokines under ArtinM stimulation, although the required concentration to induce IL-2 production was 16-fold higher than that necessary to trigger a similar response in murine T cells. In addition, the cytokine production by ArtinM-stimulated Jurkat T cells was not accompanied by cell growth, as demonstrated by the reduced frequency of viable cells after stimulation, which occurred in a dose-dependent manner. A better understanding of the growth of Jurkat T cells under stimulation with ArtinM at different concentrations (0.009–1.25 μg/mL) was obtained by performing the PDL assay for 18 days. The results demonstrated the cumulative growth-inhibitory effect of ArtinM on Jurkat T cells. Therefore, the mitogenic activity of ArtinM on murine CD4^+^ and CD8^+^ T cells was not reproduced in the Jurkat T cell line.

The glycocalyx *O*-glycans and/or *N*-glycans regulate the death of Jurkat T cells induced by lectins having distinct glycan-binding specificities, which can trigger apoptosis via caspase-dependent and/or -independent pathways [50,77,78,79,80,81,82,83]. We verified that ArtinM promotes the apoptosis of Jurkat T cells, as demonstrated by Annexin V staining and altered cell cycle progression. The mitochondrial apoptotic pathway may be responsible for the growth-inhibitory effect of ArtinM on Jurkat T cells, since mitochondrial activity decreased following ArtinM stimulation. In addition, the pretreatment of Jurkat T cells with Z-VAD decreased the ArtinM-induced apoptosis, indicating that ArtinM-induced apoptosis involves a caspase-dependent pathway. 

The signaling pathways that regulate the cell cycle are known to depend on protein kinases (MAPKs, PKC, and PTKs) to maintain the growth of Jurkat T cells [40,42,84]. Thus, the inhibition of these signaling molecules may alter the apoptotic response of Jurkat T cells. We verified that PKC, JNK, ERK, and p38 MAPK inhibitors did not alter the apoptotic effect of ArtinM on Jurkat T cells, while the PTKs inhibitor reduced the apoptosis of Jurkat T cells induced by ArtinM. Based on the findings that ArtinM exerts a cytotoxic effect on the Jurkat human leukemic T cell line and also mediates the cell death of a human myeloid leukemia cell line (NB4) [47], we postulate that ArtinM may warrant consideration as a potential candidate for the design of a novel anti-cancer therapy. This hypothesis stimulates us to deeply investigate the ArtinM effects in several cancer cell lines, under variable experimental conditions, as well as by using in vivo models of neoplastic diseases. Indeed, this approach was recently adopted to investigate the ArtinM effect on the process of hepatocarcinogenesis [85].

Our work shows that ArtinM has a mitogenic effect on murine CD4^+^ and CD8^+^ T cells, and these lectin-stimulated cells showed an immunological profile compatible with that required to confer protection against intracellular pathogens. Otherwise, the effect of ArtinM on a human leukemic T cell line was prominently cytotoxic. Therefore, the present study demonstrates a novel manner by which ArtinM may exert its immunomodulatory activity, i.e., by interacting with glycotargets on CD4^+^ and CD8^+^ T cells. In addition, this work provides new perspectives regarding the cytotoxic effect exerted by ArtinM on human leukemia cells.

## 4. Materials and Methods

### 4.1. Ethics Statement

The Committee of Ethics in Animal Research of the College of Medicine of Ribeirão Preto at the University of São Paulo approved the animal studies, Protocol no. 082/2012 (30/07/2012), which were conducted in accordance with the Ethical Principles in Animal Research adopted by the Brazilian College of Animal Experimentation.

### 4.2. Animals

Male C57BL/6 mice at 6–8 weeks of age were used in this study. These mice were acquired from the animal house of the Campus of Ribeirão Preto, University of São Paulo, Ribeirão Preto, São Paulo, Brazil. Animals were housed in the animal facility of the Molecular and Cellular Biology Department of the Faculty of Medicine of Ribeirão Preto, University of São Paulo, under optimized hygienic conditions.

### 4.3. Lectins

ArtinM was purified as previously described [25] from the saline extract of *Artocarpus heterophyllus* (jackfruit) seeds via affinity chromatography on sugar columns. Concanavalin A (ConA) from *Canavalia ensiformis* was purchased from Sigma-Aldrich (St. Louis, MO, USA). Morniga M from black mulberry (*Morus nigra*) bark was kindly provided by Els J. M. Van Damme (Department of Molecular Biotechnology, Laboratory of Biochemistry and Glycobiology, Ghent University, Ghent, Belgium). Before use, preparations of lectins were incubated for 30 min at 37 °C with polymyxin B solution (50 μg/mL) (Sigma-Aldrich) to neutralize any contamination with endotoxin.

### 4.4. Cell Suspensions

The suspensions of spleen cells obtained from mice were prepared as reported by da Silva et al. [24]. The obtained cell suspensions were used to isolate CD4^+^ and CD8^+^ T cells using the Isolation Kit II from Miltenyi Biotec (Auburn, CA, USA), according to the manufacturer’s instructions. The negatively selected cells were stained with anti-CD3 phycoerythrin (PE) and anti-CD4 fluorescein isothiocyanate (FITC) or anti-CD8 FITC antibodies (BD Biosciences, San Jose, CA, USA) and analyzed by flow cytometry (Guava^®^ easyCyte, Millipore, Billerica, MA, USA). Purity grades of 94–96% were achieved.

The Jurkat E6.1 human acute leukemia T cell line was routinely grown in advanced Roswell Park Memorial Institute 1640 medium (Gibco^®^, Life Technologies, Carlsbad, CA, USA) supplemented with 10% fetal bovine serum, 2 mM l-glutamine, 2500 mg/L glucose, 10 mM HEPES, and antibiotics in a humidified 5% CO_2_ atmosphere at 37 °C. Cell pellets were obtained by centrifugation at 300× *g* for 7 min at 4 °C. The cell concentration was maintained as recommended by the ATCC cell biology collection, a protocol that was successful adopted by several authors [50,77,80,82].

### 4.5. ArtinM Binding on T Cell Surface

Suspensions of CD4^+^, CD8^+^, and Jurkat T cells were fixed in 3% formaldehyde in phosphate-buffered saline (PBS) for 30 min at room temperature and then incubated with 1% glycine in PBS for 20 min. After two washes with PBS, the cells (2 × 10^6^/mL) were incubated with biotinylated ArtinM (concentrations specified in the figure legends) that had been pre-incubated for 40 min with either mannotriose (2 mM), lactose (20 mM), or medium alone. After further washes with PBS, the amount of biotinylated ArtinM bound on the surface of the cells was quantified via reaction with streptavidin-FITC (5 μg/mL; Life Technologies) for 40 min. Fluorescence staining was analyzed by flow cytometry (Guava^®^ easyCyte) and the percentage of stained cells was determined by the mean fluorescence intensity (MFI).

In the case of Jurkat T cells, they were incubated with biotinylated ArtinM in the presence of lectins with different carbohydrate-binding specificities (Concanavalin A [ConA], Phytohemagglutinin [PHA]-E, PHA-L, *Sambucus nigra* lectin [SNA], *Ulex europaeus* agglutinin [UEA], Morniga M, and Jacalin; each at 40 μg/mL). The amount of bound biotinylated ArtinM on the surface of the cells was detected by flow cytometry.

### 4.6. Cytokines Measurement and 3-(4,5-Dimethylthiazol-2-yl)-2,5-Diphenyltetrazolium Bromide (MTT) Assay

CD4^+^ (1.5 × 10^6^/mL), CD8^+^ (1.5 × 10^6^/mL), and Jurkat (2 × 10^5^/mL) T cells were cultured for 24 or 48 h under stimulation with ArtinM (concentrations specified in the figure legends), ConA (5 μg/mL), phorbol 12-myristate 13-acetate (PMA, 50 ng/mL; Sigma-Aldrich) plus ionomycin (1 μM; Sigma-Aldrich), or As_2_O_3_ (3 μM; Sigma-Aldrich). The cells were centrifuged at 300× *g* for 10 min at room temperature and the supernatants were harvested to determine the concentrations of IL-2, IFN-γ, and IL-1β via enzyme-linked immunosorbent assays (ELISAs) (OptEIA™; BD Biosciences), according to the manufacturer’s instructions.

The mitochondrial activity of the cells was determined after the reduction of MTT (Sigma-Aldrich) to produce formazan crystals [86]. The procedures were performed as described by da Silva et al. [24]. Mitochondrial activity was expressed as the absorbance relative to that of the negative control as a percentage value.

### 4.7. Cell Proliferation Assay

CD4^+^ and CD8^+^ T cells (1.5 × 10^6^/mL) were distributed in 96-well microplates and incubated for 48 h at 37 °C with ArtinM (1.25 μg/mL), ConA (5 μg/mL), or medium alone. The cells were treated with tritiated thymidine ([_3_H]-TdR; Amersham Bioscience, Boston, MA, USA) at 0.5 μCi/well. Cell proliferation was assessed by measuring [_3_H]-TdR incorporation and the results were expressed in counts per minute (CPM).

### 4.8. Flow Cytometry Analysis

To assess the expression of CD25, CD95, and CD69 on CD4^+^ and CD8^+^ T cells, the cells (1.5 × 10^6^/mL) were stimulated for 24 or 48 h at 37 °C with ArtinM (1.25 μg/mL) or PMA (50 ng/mL) plus ionomycin (1 μM), then washed and incubated with Fc block (10 μg/mL) for 30 min. Afterwards, the cells were washed again and incubated for 45 min with anti-CD25 PE (10 μg/mL, clone 3C7; BD Biosciences), anti-CD95 FITC (10 μg/mL, clone Jo2; BD Biosciences), or anti-CD69 FITC (10 μg/mL, clone H1.2F3; BD Biosciences). The cells were then washed with PBS and resuspended in PBS containing 0.5% formaldehyde. The frequency of fluorescent cells was analyzed by flow cytometry (Guava^®^ easyCyte).

The frequency of dead cells was assessed by analyzing the Annexin V binding and propidium iodide (PI) incorporation by CD4^+^, CD8^+^, and Jurkat T cells. They were incubated with ArtinM (concentrations specified in the figure legends), staurosporine (1 μM), As_2_O_3_ (3 μM), or medium alone and then stained with Annexin V-FITC (for 40 min) and PI (for an additional 5 min). The fluorescence emission due to the reaction of cells with Annexin V and PI was quantified using a Guava^®^ easyCyte flow cytometer.

### 4.9. Quantitative Reverse Transcription Polymerase Chain Reaction for the Detection of Tbet and Gata3 Transcripts in CD4^+^ T Cells

CD4^+^ T cells (1.5 × 10^6^ cells/mL) from C57BL/6 mice were distributed in 48-well microplates and incubated at 37 °C. After 8 h incubation under stimulation with ArtinM (1.25 μg/mL), IL-4 (50 ng/mL; PeproTech, Rock Hill, NJ, USA), or IL-12 (50 ng/mL; PeproTech) plus IFN-γ (30 ng/mL; PeproTech), total RNA was isolated from CD4^+^ T cells using TRIzol^®^ Reagent (Life Technologies), according to the manufacturer’s instructions. The reverse transcription of RNA into cDNA was performed as reported by da Silva et al. [37]. qRT-PCR was performed in 15 μL reactions using SYBR Green (Applied Biosystems/Life Technologies, Carlsbad, CA, USA). qRT-PCR was performed with the 7500 Real-Time PCR System (Applied Biosystems) using the following conditions: 50 °C for 2 min, 95 °C for 10 min, and 40 cycles of 95°C for 15 s/60 °C for 1 min. Gene expression was quantified using the ΔΔ*C*_t_ method and normalized to *Gapdh* and β-actin (*Actb*) expression. The specificity of the amplification was determined by the melting curves analysis. Polymerase chain reaction primers utilized were: *Actb* (F: 5′-AGCTGCGTTTTACACCCTTT-3′/R: 5′-AAGCCATGCCAATGTTGTCT-3′); *Gapdh* (F: 5′-TGCCCCCATGTTTGTGATG-3′/R: 5′-TGTGGTCATGAGCCCTTCC-3′); *Tbet* (F: 5′-CACTAAGCAAGGACGGCGAA-3′/R: 5′-CCACCAAGACCACATCCAC-3′); and *Gata3* (F: 5′-AAGAAAGGCATGAAGGACGC-3′/R: 5′-GTGTGCCCATTTGGACATCA-3′).

### 4.10. Assays for Functional and Binding Competition between Anti-CD3 Antibody and ArtinM

The purified CD8^+^ and CD4^+^ T cells (1 × 10^6^/mL) were fixed and incubated with or without ArtinM (25 μg/mL) for 40 min at 4 °C. Afterwards, the cells were incubated with 145-2C11 monoclonal antibody (conjugated to PE) or 17A2 monoclonal antibody (conjugated to FITC) (both 10 μg/mL; BD Biosciences), which specifically bind to the CD3ε and CD3εγ chains, respectively. After incubation with anti-CD3 antibody for 40 min at 4 °C, the cells were washed with PBS and the percentage of cells labeled with anti-CD3 antibody was determined using flow cytometry (Guava^®^ EasyCyte). Cells that were not stained with anti-CD3 antibody were incubated with IgG isotype control antibodies (FITC or PE) to use as controls.

The functional competition assay was performed using isolated CD8^+^ T cells (1.5 × 10^6^/mL), which were incubated with 17A2 monoclonal antibody against CD3 (8 μg/mL; eBioscience, San Diego, CA, USA) or IgG isotype control (A19-3, 8 μg/mL; BD Biosciences) for 40 min at 4 °C. Afterwards, the cells were cultured in 96-well microplates for 48 h at 37 °C under stimulation with ArtinM (1.25 μg/mL), PMA (50 ng/mL) plus ionomycin (1 μM), or medium alone. The culture supernatants were used for the measurement of IL-2 by ELISA.

### 4.11. Population Doubling Level (PDL) Assay

Jurkat T cells (1 × 10^5^ cells/mL) were distributed in 96-well microplates and incubated at 37 °C for 72 h in the presence of ArtinM (0.009–1.25 μg/mL), As_2_O_3_ (1.5 μM) as a positive control, or medium alone as the negative control. The cell suspension was adjusted to a concentration of 1 × 10^5^ cells/mL, distributed in 96-well microplates and stimulated for 72 h as specified above, then the cells were incubated for 18 days. To determine the cumulative PDL for each condition, the following equation was used: 2 × [log(*C_f_*/*C_i_*)] + *PDL_p_*. The PDL was calculated every 72 h by considering the following parameters: final cell concentration (*C_f_*); initial cell concentration (1 × 10^5^ cells/mL; *C_i_*); and previous PDL (*PDL_p_*).

### 4.12. Caspase Inhibition

Jurkat T cells (2 × 10^5^/mL) were incubated with a broad-spectrum caspase inhibitor (Z-VAD, 75 μM; purchased from Sigma-Aldrich) for 3 h. Subsequently, the cells were stimulated for 24 h with ArtinM (10 or 20 μg/mL), As_2_O_3_ (3 μM), or medium alone, and then reacted with Annexin V-FITC and PI. The frequencies of Annexin V/PI double-stained cells and Annexin V single-stained cells were analyzed by flow cytometry.

### 4.13. Treatment of Jurkat T Cells with Inhibitors of Cell Signaling Molecules

Jurkat T cells (2 × 10^5^/mL) were distributed in 96-well microplates and treated with the following pharmacological inhibitors at concentrations between 5–40 μM: Genistein (PTKs inhibitor), PD98059 (ERK inhibitor), SB202190 (p38 MAPK inhibitor), H-7 (PKC inhibitor), and SP600125 (JNK inhibitor) were purchased from Sigma-Aldrich. After 210 min incubation, the Jurkat T cells were stimulated for 48 h with ArtinM (20 μg/mL), As_2_O_3_ (3 μM), or medium alone. Then, the Jurkat T cells were analyzed by flow cytometry to determine Annexin V-FITC binding and PI incorporation. The results were expressed as the frequency of Annexin V/PI- or Annexin V-stained cells as a percentage value.

### 4.14. Statistical Analysis

The results are presented as means ± standard error of the mean and all data were analyzed using Prism (GraphPad Software, La Jolla, CA, USA). Statistical evaluation of the differences among group means was performed using one-way analysis of variance followed by Bonferroni’s multiple comparison test, and nonparametric test the comparisons between groups were performed using Dunn’s multiple comparison test. Differences of *p* < 0.05 were considered statistically significant.

## Supplementary Materials

Supplementary materials can be found at www.mdpi.com/1422-0067/18/7/1400/s1.
