# Supplementary material for: ArtinM Mediates Murine T Cell Activation and Induces Cell Death in Jurkat Human Leukemic T Cells
Source: Int J Mol Sci. 2017 Jun 30;18(7):1400. doi: 10.3390/ijms18071400 (PMC5535893; doi:10.3390/ijms18071400)
Supplement: Supplementary file 1 [file ijms-18-01400-s001.pdf]

ArtinM mediates murine T cell activation and induces cell death in Jurkat human leukemic T cells

Thiago Aparecido da Silva, Patrícia Kellen Martins Oliveira-Brito, Thiago Eleutério Gonçalves, Patrícia Edivânia Vendruscolo, Maria Cristina Roque-Barreira *

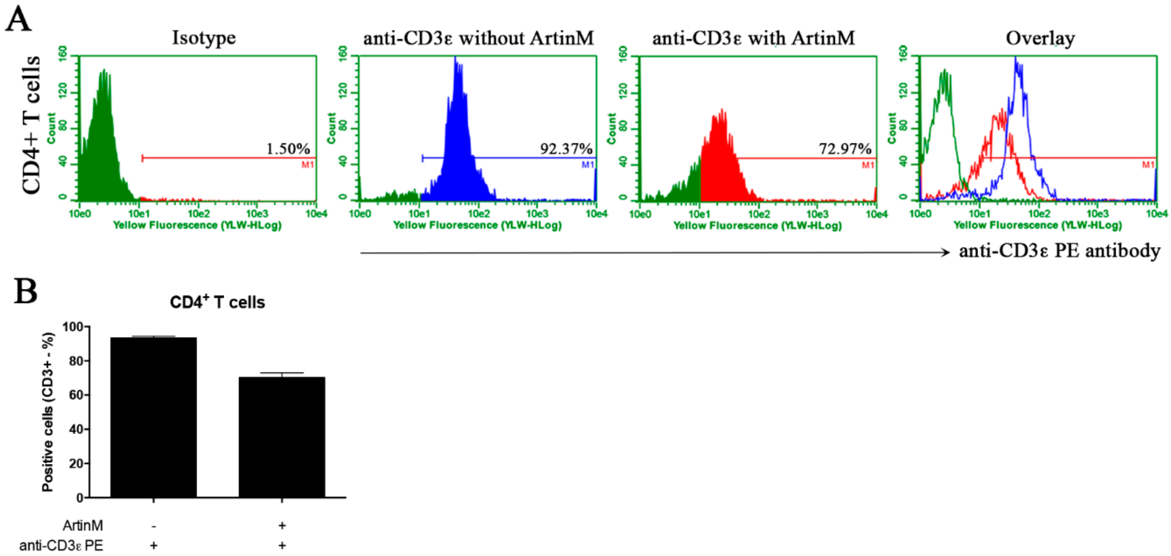

Figure S1. The competition between ArtinM and anti-CD3ε antibody for binding on CD4+ T cells. Purified CD4+ T cells (1 × 10⁶/mL) were fixed and incubated with or without ArtinM (25 μg/mL) for 40 min. Afterwards, the cells were incubated for 40 min with 145-2C11 monoclonal antibody (conjugated to PE), which is specific to the CD3ε chain. (**A**) The labeled cells were analyzed by flow cytometry. The histograms represent the percentage of cells that were positive for anti-CD3 antibody after pre-incubation with or without ArtinM, and the overlay represents all these conditions; (**B**) the graphic represents the values in replicates of positive cells for anti-CD3 antibody in the presence or absence of ArtinM. The results are expressed as means ± SEM.

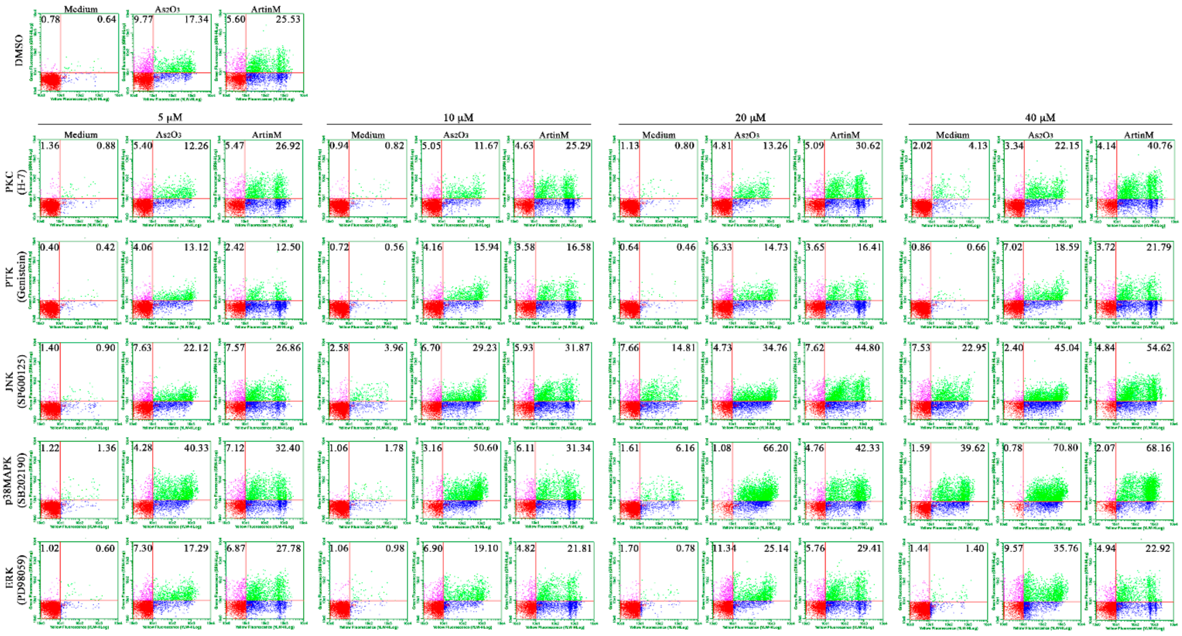

Figure S2. Dot plots of Annexin V and/or PI positive Jurkat T cells incubated with pharmacological signaling pathway inhibitors and stimulated with or without ArtinM. Jurkat T cells (2 × 10⁵/mL), as described in Figure 8, were assayed in the presence or absence of inhibitors against PKC, JNK, p38

MAPK, ERK, and PTKs at different concentrations (5–40 µM). Then, the cells were stimulated with ArtinM (20 µg/mL), As_2O_3 (3 µM), or medium alone for 48 h. Annexin V-FITC binding and PI incorporation were analyzed by flow cytometry and the dot plots show the percentage of positive cells for Annexin V/PI, Annexin V, or PI.

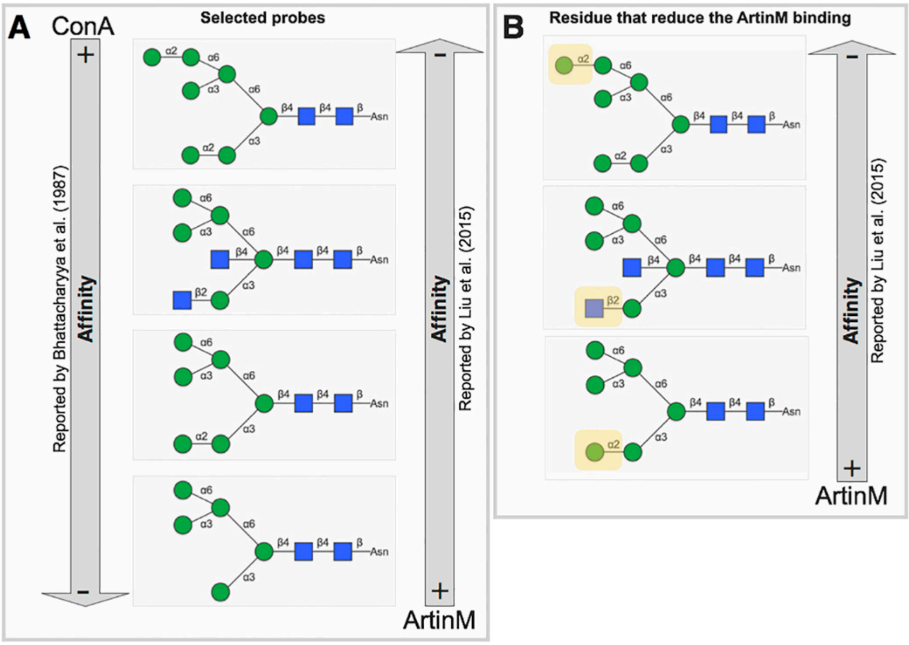

Figure S3. Analysis of carbohydrate-recognition by ConA and ArtinM. (**A**) The attribution of ConA affinity for each sugar structure is based on the analyses by Bhattacharyya et al. [52]. The selected structures were analyzed for ArtinM-binding using a glycan microarray system [49]. (**B**) The evaluation of ArtinM binding to 255 glycans distributed in a microarray platform allowed the identification of which residues in well-recognized probes for ConA reduced the ArtinM binding (**yellow**). Blue squares and green circles represent Mannose and N-acetylglucosamine, respectively.
